# Prediction and validation of anoikis-related genes in neuropathic pain using machine learning

**DOI:** 10.1371/journal.pone.0314773

**Published:** 2025-02-27

**Authors:** Yufeng He, Ye Wei, Yongxin Wang, Chunyan Ling, Xiang Qi, Siyu Geng, Yingtong Meng, Hao Deng, Qisong Zhang, Xiaoling Qin, Guanghui Chen

**Affiliations:** 1 Department of Massage, The First Affiliated Hospital of Guangxi University of Chinese Medicine, Nanning, China; 2 Department of Rehabilitation Medicine, The First Affiliated Hospital of Guangxi University of Chinese Medicine, Nanning, China; 3 Graduate School, Guangxi University of Chinese Medicine, Nanning, China; 4 Graduate School, Henan University of Chinese Medicine, Zhengzhou, China; 5 Second Clinical Medical College, Henan University of Chinese Medicine, Zhengzhou, China; 6 Medical College, Guangxi University, Nanning, China; 7 School of Pharmacy, Guangxi University of Chinese Medicine, Nanning, China; Nankai University, CHINA

## Abstract

**Background:**

Neuropathic pain (NP) can be induced by a variety of clinical conditions, such as spinal cord injury, lumbar disc herniation (LDH), lumbar spinal stenosis, diabetes, herpes zoster, and spinal cord tumors, and inflammatory stimuli. The pathogenesis of NP is extremely complex. Specifically, in LDH, the herniated nucleus pulposus exerts mechanical pressure on nerve roots, triggering local inflammation and consequent NP. Anoikis, a special form of programmed cell death, is closely related to the progression of NP. In this study, we sought to clarify the molecular characteristics of anoikis-related genes in NP, providing novel insights for the diagnosis and treatment of NP.

**Methods:**

We screened NP-related genes based on the GSE124272 dataset and obtained 439 anoikis-related genes from the GeneCards database. Through Least Absolute Shrinkage and Selection Operator (LASSO) and Support Vector Machine (SVM) machine learning algorithms, six key hub genes were identified: hepatocyte growth factor (*HGF*), matrix metalloproteinase 13 (*MMP13*), c-abl oncogene 1, non-receptor tyrosine kinase (*ABL1*), elastase neutrophil expressed (*ELANE*), fatty acid synthase (*FASN*), and long non-coding RNA (*Linc00324*). Functional enrichment analyses, including Gene Ontology (GO) and Kyoto Encyclopedia of Genes and Genomes (KEGG), alongside Gene Set Enrichment Analysis (GSEA) and immune infiltration analysis, were performed on these hub genes. Additionally, transcription factors and potential therapeutic drugs were predicted. We also used rats to construct an NP model and validated the analyzed hub genes using hematoxylin and eosin (H&E) staining, real-time polymerase chain reaction (PCR), and Western blotting assays.

**Results:**

Our data indicated that anoikis-related genes have diagnostic value in NP patients, as confirmed by experimental results. Moreover, this study elucidated the role of these genes in immune infiltration during the pathogenesis of NP and identified potential therapeutic drugs targeting these key genes.

**Conclusion:**

This study further explores the pathogenesis of NP and provides certain reference value for developing targeted therapeutic strategies, thereby improving NP management.

## Introduction

Neuropathic pain (NP) arises from diseases affecting the peripheral or central nervous system, especially the somatosensory nervous system [1]. It has been established that the protrusion of the nucleus pulposus can mechanically irritate and compress nerve roots, leading to localized inflammation and consequent NP [2]. Epidemiological data indicate that NP affects between 6.9% and 10% of the global population [3]. The ineffectiveness of current NP prevention and treatment strategies impacts global health [4], highlighting the urgent need for safe and efficient interventions due to the complex and poorly understood pathophysiology of NP.

Anoikis is a type of programmed cell death distinguished by the detachment of cells from the extracellular matrix, leading to apoptosis following the loss of contact with neighboring cells or the matrix itself [5]. Recent studies have associated anoikis-related genes with ophthalmic diseases, inflammatory bowel disease, and Parkinson’s disease [6–8]. Furthermore, anoikis has been linked to neuronal death in the hippocampus [9] and observed to impair neuronal metabolism through neuronal and astrocyte pathways, leading to neurodegenerative diseases [10]. These observations imply an association between neuronal cell death and anoikis.

Neuronal cell death has been confirmed as a contributing factor to the onset of NP [11]. The progression of NP is influenced by various cellular processes, such as apoptosis, autophagy, mitophagy, ferroptosis, pyroptosis, necroptosis, and phagocytosis [12]. Therefore, it is plausible that anoikis may notably impact the progression of NP. While previous studies have linked anoikis to various diseases, its specific role in NP remains unexplored. Elucidating this relationship could provide novel insight into NP pathogenesis and treatment. Therefore, this study aims to examine the molecular characteristics of anoikis-related genes in NP, with the goal of providing new perspectives for NP diagnosis and treatment.

## Materials & methods

### Data acquisition

We analyzed the gene expression profiles of patients with LDH-related NP, which were obtained from the GSE124272 dataset on the GPL21185 platform, accessed *via* the Gene Expression Omnibus (GEO) database [13,14]. This dataset contained eight patients with LDH-related NP and eight volunteers without NP.

Anoikis-related genes were obtained from the GeneCards database (https://www.genecards.org/) [15]. A comprehensive set of 917 anoikis-related genes was (as of September 1, 2023). Based on the criteria established by Chen Y [16], genes with a score >0.7 were selected, resulting in 439 genes obtained for subsequent analysis.

### Pre-test of anoikis-related genes

A preliminary exploration of anoikis-related genes on human chromosomes was conducted using the "RCircos" package in R software (version 4.3.1) [17]. Key genes were then visualized by creating a circular plot of human chromosomes depicting anoikis-related genes.

### Identification and functional analysis of anoikis-related genes

Anoikis-related genes from the gene expression profiles of patients with LDH-related NP were extracted using the "limma" package in R [18]. Differential expression analysis was conducted to identify genes with altered expression in patients with LDH-related NP compared with those in healthy volunteers. Correlations between differentially expressed genes (DEGs) were analyzed using "corrplot" and "circlize" packages in R. Gene Ontology (GO) and Kyoto Encyclopedia of Genes and Genomes (KEGG) enrichment analyses were performed on DEGs using the "enrichplot", "ggplot2", and "org.Hs.eg.db" packages in R.

### Screening of key anoikis-related genes using Support Vector Machine (SVM) algorithms

We screened for key anoikis-related genes associated with LDH-related NP using SVM algorithms [19]. Preliminary screening was performed using Least Absolute Shrinkage And Selection Operator (LASSO) regression [20] with 1000-fold cross-validation (R package "glmnet"). Recursive feature elimination (RFE) with SVM was then used to identify key genes, minimizing overfitting (R package "e1071"). Finally, overlapping genes identified by the LASSO regression and SVM were considered key anoikis-related genes associated with LDH-related NP (R package "VennDiagram").

### Performance validation of hub genes among anoikis-related genes

The diagnostic performance of hub genes was validated using receiver operating characteristic (ROC) curve analysis [21] ("glmnet" and "pROC" packages in R).

### Gene set enrichment analysis (GSEA) and immunoassay of hub genes

GSEA differs from GO enrichment analysis in that it is an analytical method with an unsupervised algorithm based on gene set enrichment [22]. We performed GSEA to explore the biological mechanisms of the hub genes among the anoikis-related genes associated with LDH-related NP. Immune cell infiltration was evaluated in patient and healthy samples using the cell-type identification by estimating relative subsets of RNA transcripts (CIBERSORT) method. Subsequently, we assessed the association between hub genes among anoikis-related genes and the infiltration of immune cells.

### Prediction of TFs and potential medications

Transcription factors (TFs) and potential medications associated with LDH-related NP were identified using the Enrichr analysis platform (https://maayanlab.cloud/Enrichr/) [23]. Key anoikis-related genes were analyzed to determine relevant TFs and therapeutic agents.

### Experimental validation of hub genes

#### Animal modeling and grouping

A total of 12 male specific pathogen-free (SPF)-grade rats weighing 280–300 g (qualification No. SYXK [Gui] 2019–0001) were provided by Hunan Silaikejingda Experimental Animal Co., Ltd. The rats were randomly assigned into a blank group or a model group, with six rats in each group. The rats in the model group underwent left lumbar 5 (L5) spinal nerve ligation, while those in the blank group were fed routinely and did not undergo modeling surgery. After 7 days, euthanasia was induced with an intraperitoneal injection of 1% pentobarbital sodium at a dose of 80 mg/kg, L4-L6 spinal cord segments and nerve roots were extracted for further analyses. The Laboratory Animal Welfare Ethics Committee of the Guangxi University of Chinese Medicine approved the study protocol (Approval No. DW20230525-101).

#### Hematoxylin and eosin (H&E) staining

H&E staining was employed to observe the morphological changes in the dorsal horn of the rat spinal cords (L4–L6). The sections were soaked in PBS, rinsed with distilled water for 30 s, and stained with hematoxylin, followed by rinsing with distilled water. A bluing step was performed, after which the sections were again rinsed with distilled water. The sections were then stained with eosin, rinsed with water, and dehydrated using a graded ethanol series. To increase transparency, the sections were treated with xylene and sealed with neutral gum. Morphological changes in the dorsal horn were examined under a microscope in three fields of view per section.

#### Real-time PCR assay

Total RNA was extracted from the L4L6 spinal cords of the rats *via* the TRIzol method, with β-actin as an internal reference gene. The concentration and quality of RNA were assessed and reverse transcription was performed using the CWbio RT Kit according to the manufacturer’s instructions. cDNA synthesis was conducted with the Reverse Transcription Kit. PCR amplification of target and internal reference gene primers was performed using a three-step PCR program: pre-denaturation at 95°C for 15 min, denaturation at 95°C for 10 s, annealing at 55°C for 30 s, and extension at 72°C for 30 s, for a total of 40 cycles. Each sample was run in triplicate. The relative mRNA expression level was calculated *via* the 2^-ΔΔCt^ method. The primer sequences are shown in Table 1.

**Table 1:** PCR primers.

Gene	Primer sequence
*HGF*	TCCCGTTGTGAAGGAGATAC
	TTCAAACTAACCATCCACCC
*ABL1*	CCCAAGCAACTACATCACCC
	GTACACCCTCCCTTCATACCG
*MMP13*	GTTGATAGACTCCGAGAAATGC
	GTTAAGTTTGTTTGGGACCATT
*ELANE*	GAGGAGGCTGTGGATCTGGATT
	GGTCTTTGGGATTGGTAAGTGGC
*FASN*	GTCCTGTTATCACCCGACTTCCT
	TGCTGAATACGACCACGCACTA
*β-actin*	TGACGTTGACATCCGTAAAGACC
	GTGCTAGGAGCCAGGGCAGTAA

### Western blotting assay

The total protein was extracted from the L4–L6 spinal cords of the rats in each group. The protein concentration was measured using the BCA kit, with β-actin as an internal reference protein. Samples were loaded into wells, electrophoresed, and wet transferred to the polyvinylidene fluoride (PVDF) membrane. The membrane was subsequently immersed in a blocking solution for 2 h, followed by incubation with primary antibodies at 4°C overnight. After washing, the membrane was incubated with a secondary antibody solution for 1 h.Visualization was performed using a chemiluminescence imaging system, and gray values were analyzed using Image J software.

### Statistical analysis

Experimental data were statistically analyzed using GraphPad Prism 9.0 software, and independent samples t-tests were employed to assess significant differences. P < 0.05 represented a statistically significant difference.

## Results

### Identification and functional analysis results of anoikis-related genes

**Fig 1:**
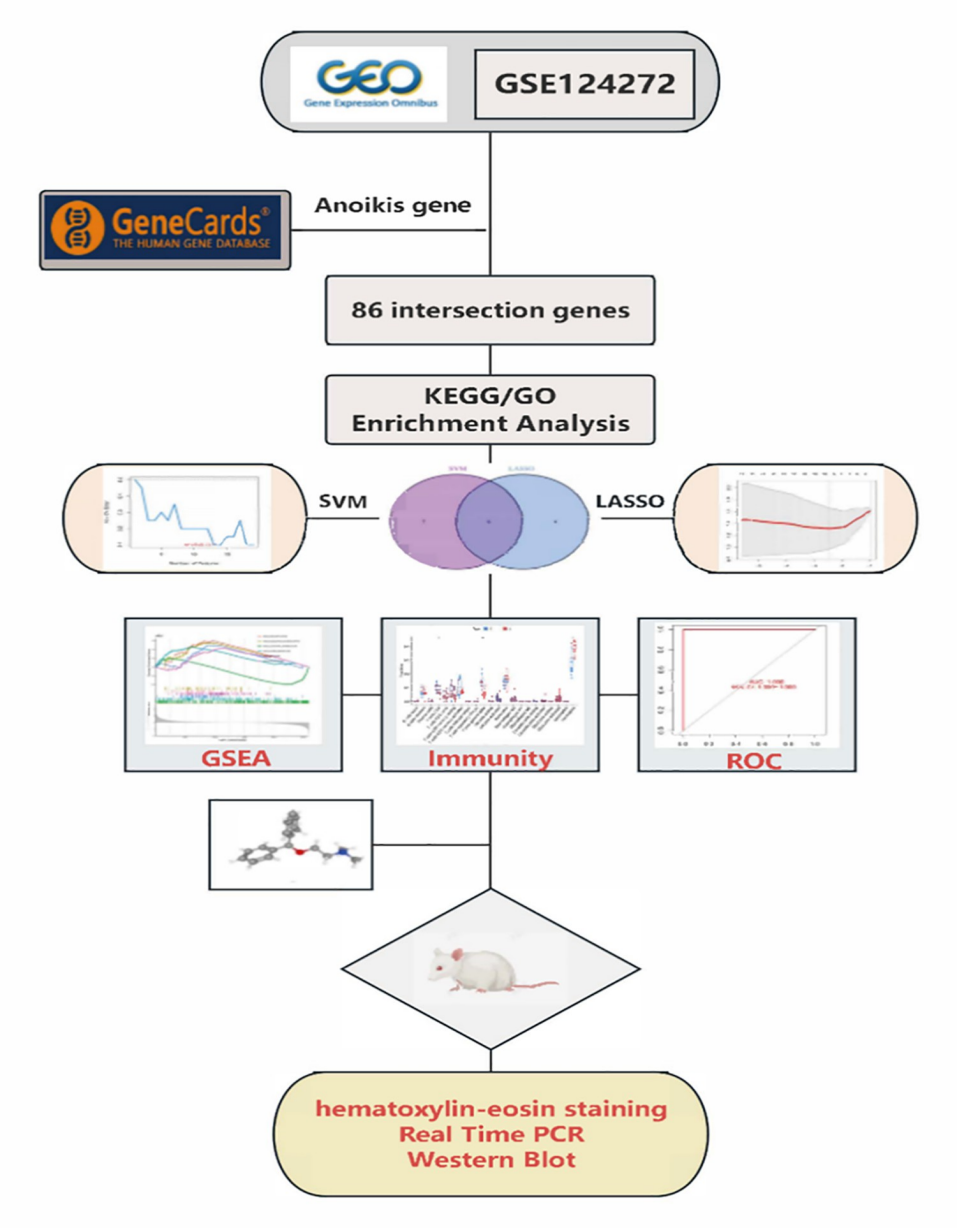
Flow chart of the experimental procedure.

**Fig 2:**
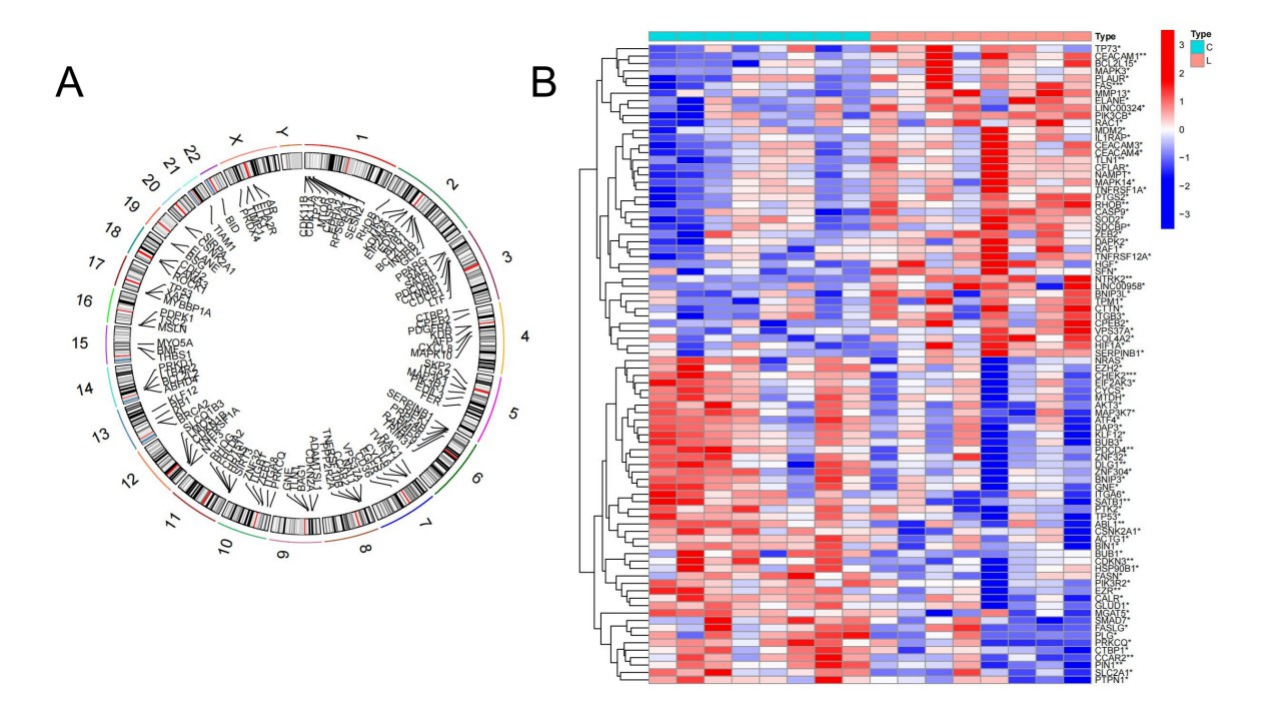
Identification of anoikis-related genes. A. A circular plot of human chromosomes of anoikis-related genes. B. A heat map of anoikis-related genes.

**Fig 3:**
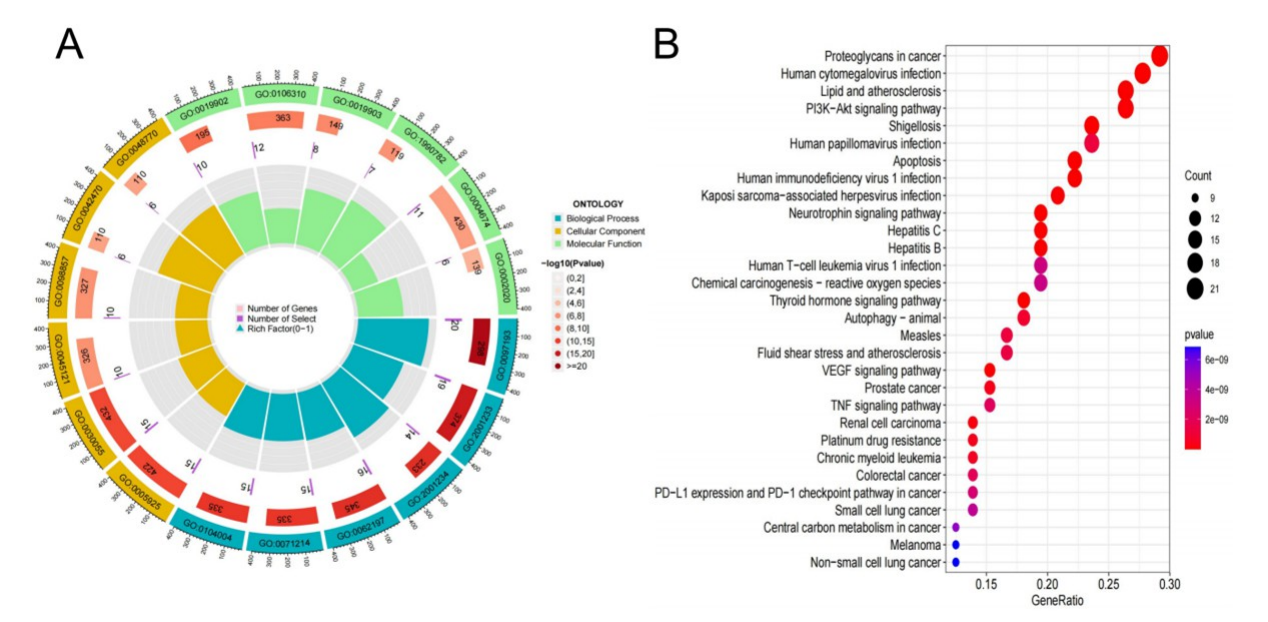
Functional analysis of anoikis-related genes. A. A circular plot of human chromosomes of anoikis-related genes. B. A heat map of anoikis-related genes.

The research workflow of this study is depicted in Fig 1. A circular plot of the human chromosomes demonstrates the specific locations of copy number variations in key anoikis-related genes on each chromosome. In this visualization, the outer circle represents the chromosomes, while the inner circle represents the important anoikis-related genes (Fig 2A). The analysis revealed that these key anoikis-related genes were distributed across all human chromosomes. The expression data of the anoikis-related genes were extracted from patients with LDH-related NP and healthy volunteers using the R package "limma". Comparative analysis identified 86 differentially expressed anoikis-related genes between the two groups. A heat map was generated to display the expression levels of these genes in each sample (Fig 2B). The data indicated that 42 DEGs were upregulated in patients with LDH-related NP, whereas 44 genes were upregulated in healthy volunteers. Functional enrichment analysis of the 86 differentially expressed anoikis-related genes indicated their involvement in several biological functions and pathways. These genes were enriched for molecular functions, such as protein serine kinase activity and protein serine/threonine kinase activity (Fig 3A). Furthermore, these genes exhibited enrichment in signaling pathways such as the PI3K-Akt pathway, VEGF pathway, and TNF pathway (Fig 3B).

### Screening of key anoikis-related genes using SVM

**Fig 4:**
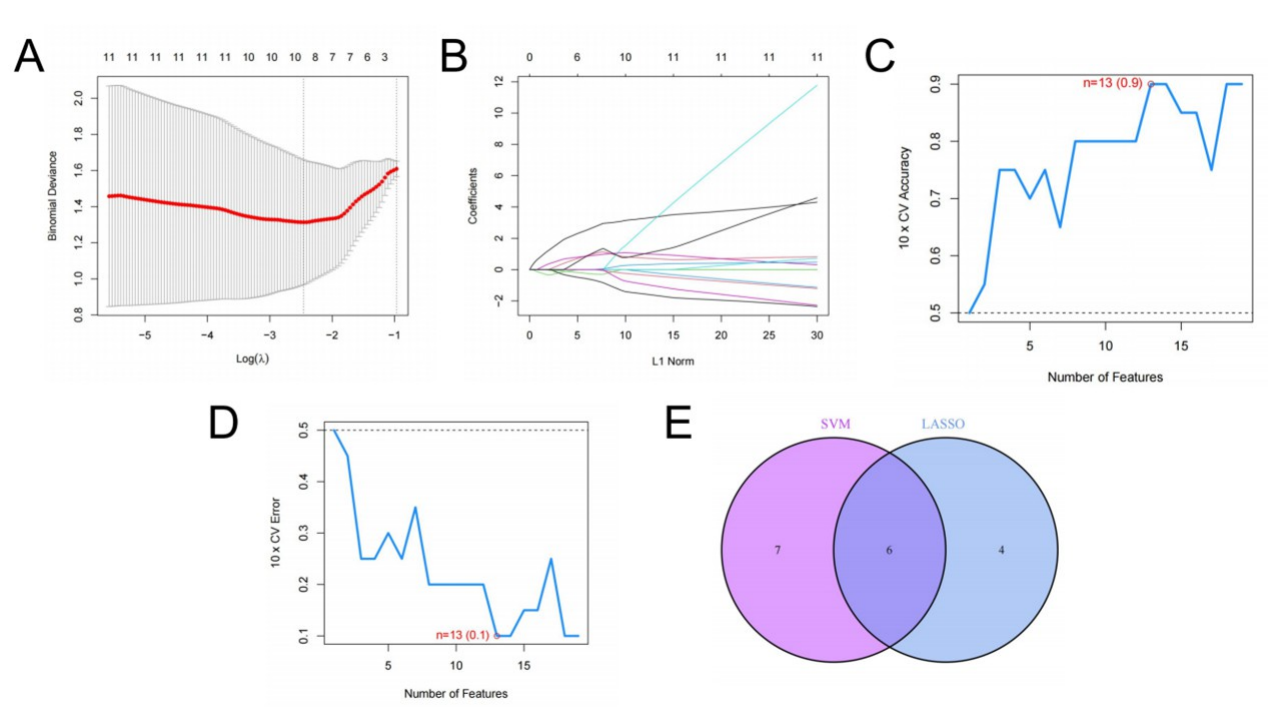
Selection of key anoikis-related genes. (A-B) Screening of key anoikis-related genes using LASSO regression. (C-D) Accuracy and error plots for screening key anoikis-related genes by SVM. (E) A Venn diagram showing the overlap of key anoikis-related genes from LASSO regression and SVM analyses.

Initially, ten key anoikis-related genes associated with LDH-related NP were identified using LASSO regression with 1,000-fold cross-validation (Fig 4A and 4B). To refine this selection and avoid overfitting, SVM analysis was performed, identifying 13 key anoikis-related genes in patients with LDH-related NP (Fig 4C and 4D). The overlapping genes from both LASSO and SVM analyses (*HGF*, *MMP13*, *ABL1*, *ELANE*, *FASN*, and *LINC00324*) were consolidated as the final key genes (Fig 4E).

### Performance validation of key anoikis-related genes

**Fig 5:**
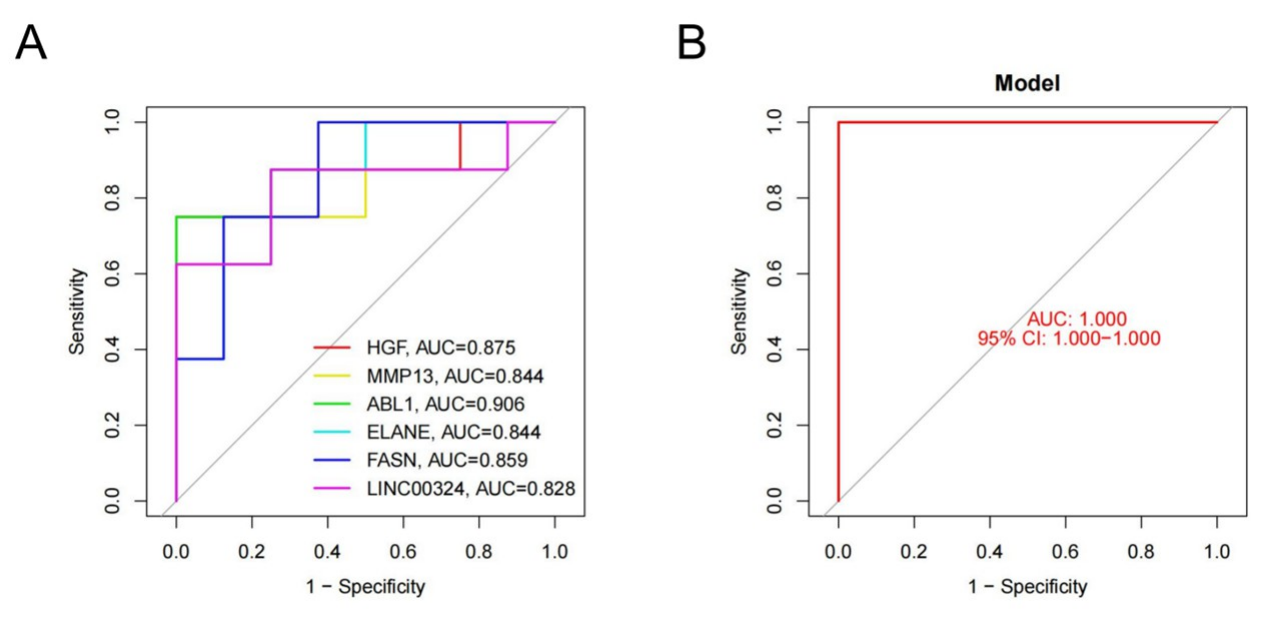
Validation of the performance of 6 key anoikis-related genes. (A) ROC curve showing the AUC value of each key anoikis-related gene. (B) ROC curve validating the performance of key anoikis-related genes.

The diagnostic value of *HGF*, *MMP13*, *ABL1*, *ELANE*, *FASN*, and *LINC00324* in patients with LDH-related NP was verified using ROC curve analysis.The results showed that all six genes exhibited significant diagnostic performance, and the area under the curve (AUC) values of each gene were as follows: *HGF* (0.875), *MMP13* (0.844), *ABL1* (0.906), *ELANE* (0.844), *FASN* (0.859) and *LINC00324* (0.828) (Fig 5A). The combined model of these six key anoikis-related genes achieved an AUC value of 1.000, suggesting excellent diagnostic accuracy for identifying patients with LDH-related NP (Fig 5B).

### GSEA and immunoassay of hub genes

**Fig 6:**
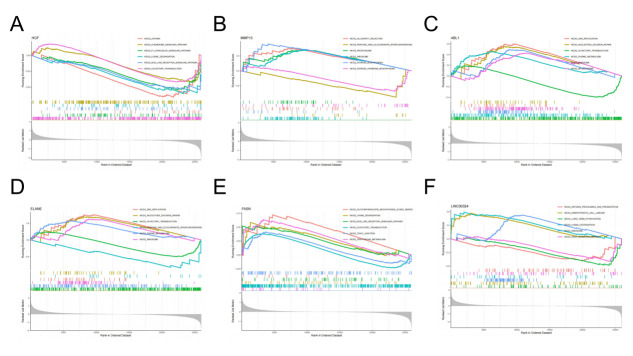
GSEA of key anoikis-related genes. (A) GSEA of *HGF*. (B) GSEA of *MMP13*. (C) GSEA of *ABL1*. (D) GSEA of *ELANE*. (E) GSEA of *FASN*. (F) GSEA of *LINC00324*.

**Fig 7:**
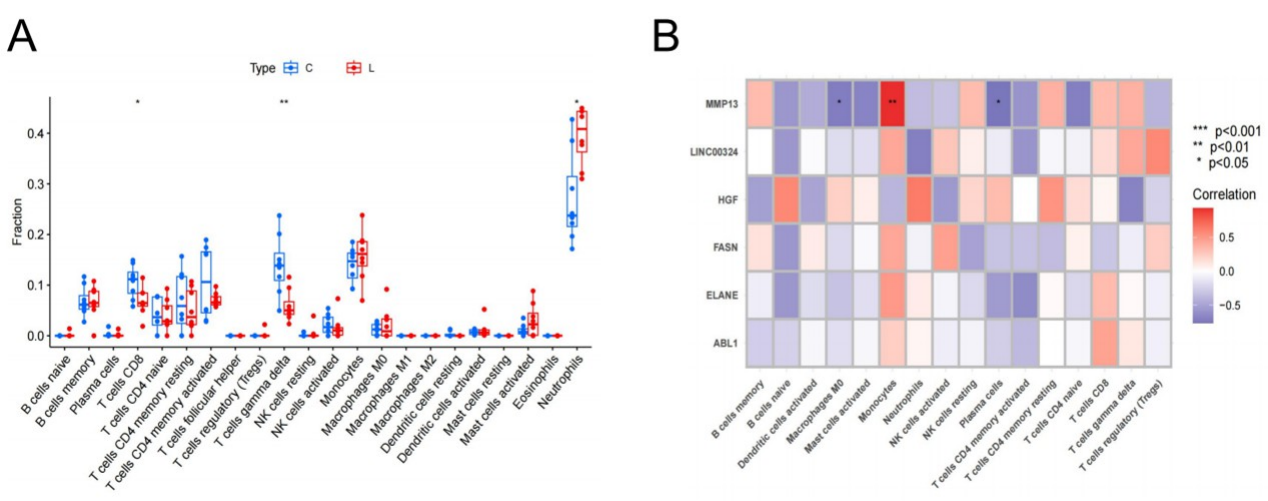
Immunoassay of key anoikis-related genes. (A) Differences in immune cell infiltration levels between patients with LDH-related NP and healthy volunteers. (B) Correlation of key anoikis-related genes with immune cell infiltration levels.

GSEA results of *HGF*, *MMP13*, *ABL1*, *ELANE*, *FASN*, and *LINC00324* suggested that these six hub genes were primarily enriched in immune-related pathways and involved in biological processes related to genetics and metabolism (Fig 6A–6F). To predict patient prognosis, we conducted immune cell infiltration analysis using the CIBERSORT algorithm. This analysis compared immune cell infiltration in patients with LDH-related NP and healthy volunteers and analyzed the correlation between immune cells and the six hub genes. Significant differences were observed in the infiltration of CD8 + T cells, γδ T cells, and neutrophils between patients with LDH-related NP and healthy volunteers (P < 0.05, Fig 7A). Further analysis revealed correlations between these hub genes and 16 types of immune cells, of which only MMP13 and Macrophages M0 were negatively correlated with plasma cells and positively correlated with monocytes (P < 0.05, Fig 7B).

### Prediction of TFs and potential medications

**Fig 8:**
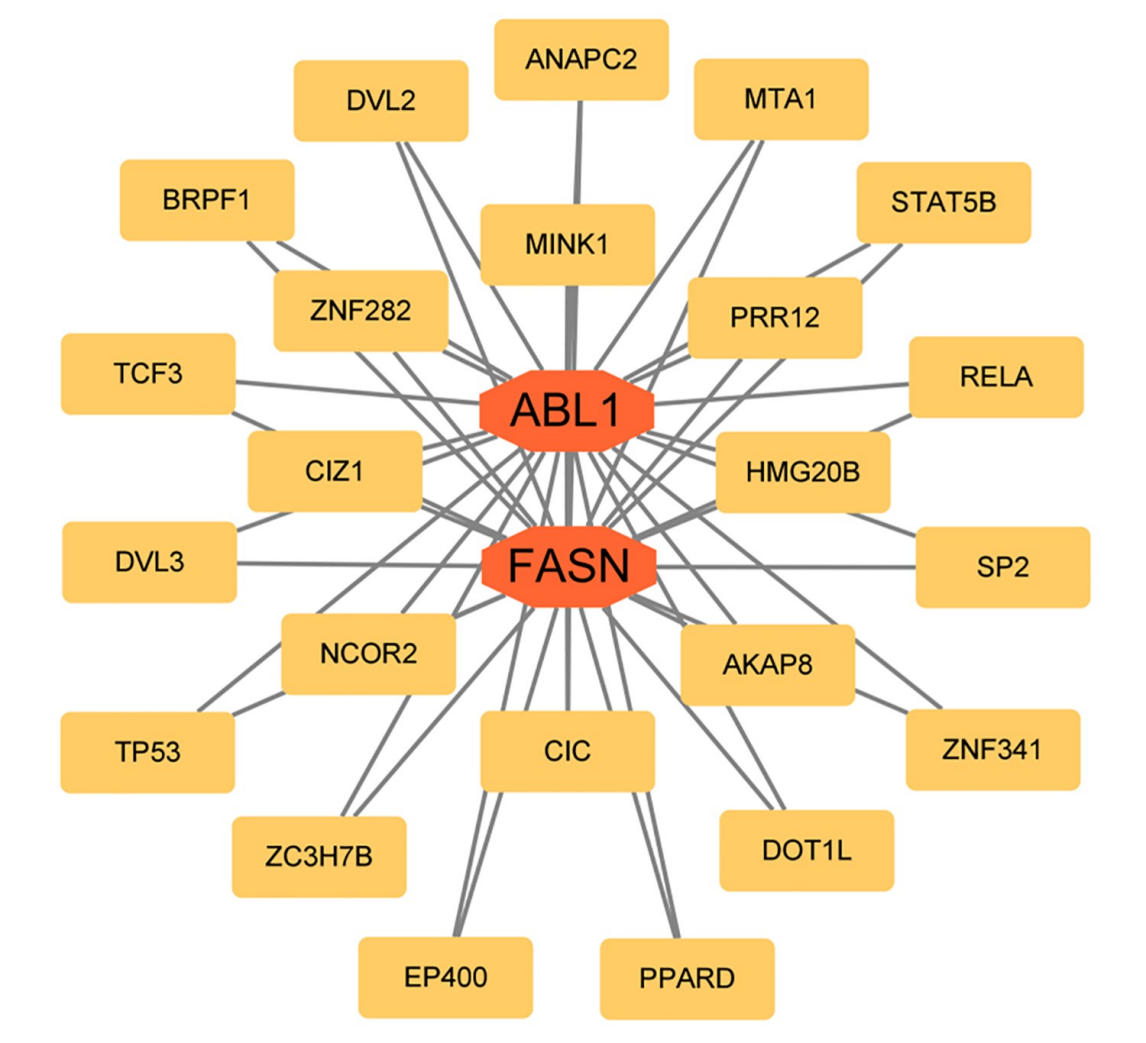
Prediction of TFs for key anoikis-related genes.

**Fig 9:**
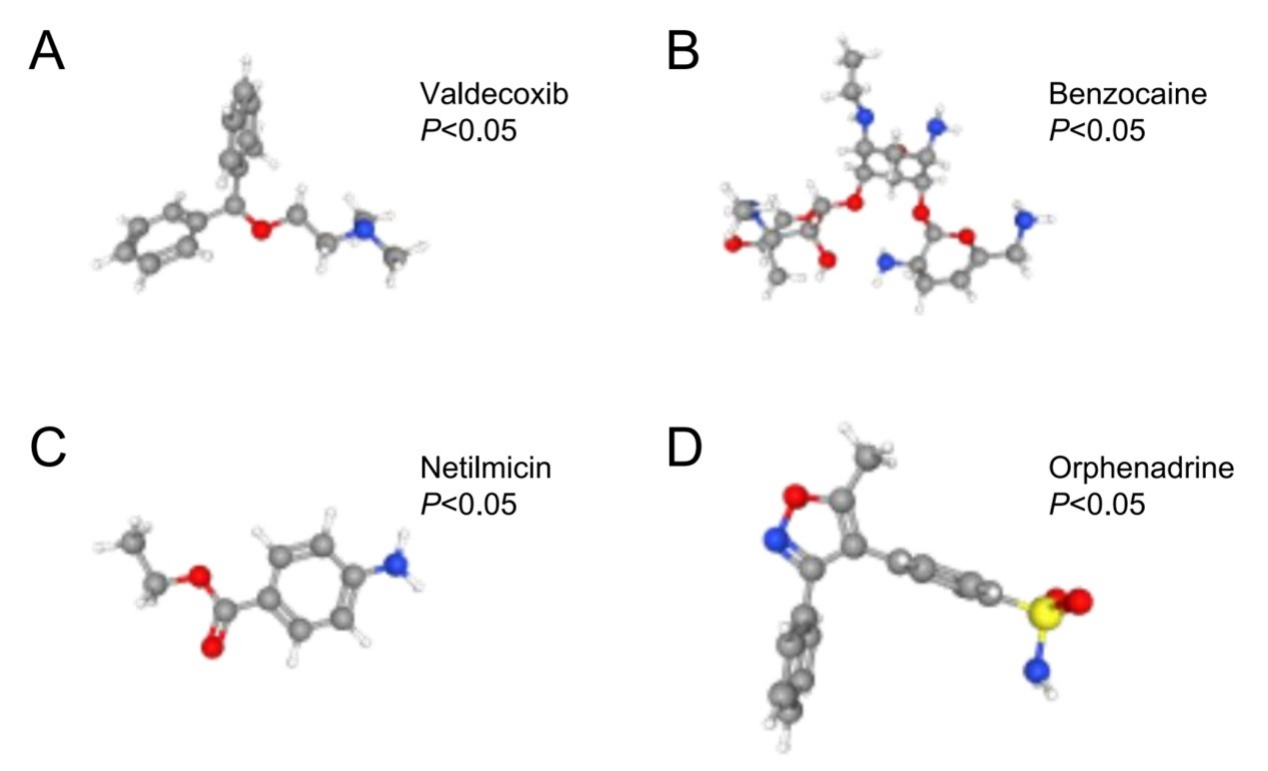
Four potential therapeutic drugs. (A) valdecoxib; (B) benzocaine; (C)netilmicin; (D) orphenadrine.

Our exploration of the Enrichr platform indicated that 23 TFs were associated with the key anoikis-related genes in the context of LDH-related NP. The key anoikis-related genes most relevant to TFs were *ABL1* and *FASN* (P < 0.05, Fig 8). In addition, we identified four potential drugs with anti-inflammatory and analgesic effects for treating LDH-related NP: valdecoxib, benzocaine, netilmicin, and orphenadrine (P < 0.05, Fig 9).

### Experimental results

**Fig 10:**
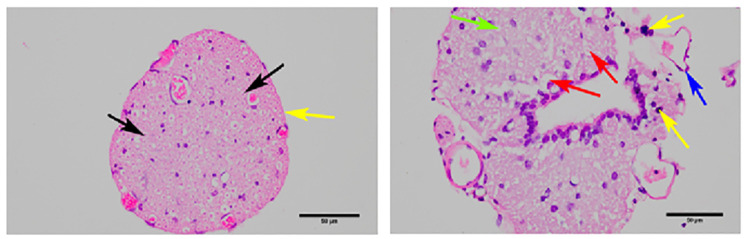
H&E staining results of rats in each group.

**Fig 11:**
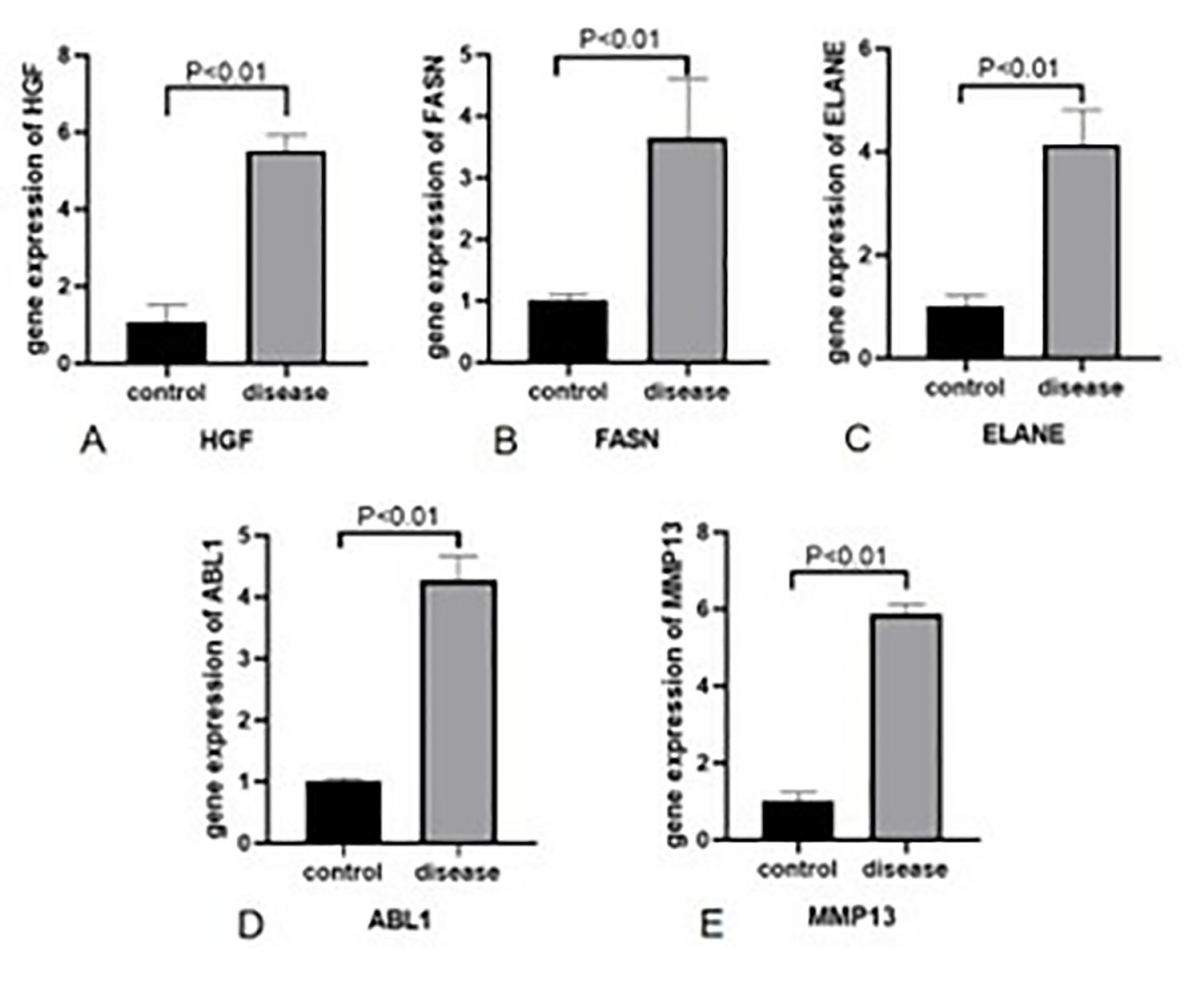
Expression levels of mRNA in the spinal cord of each group of rats. The data were analyzed using independent sample t-tests and expressed as mean P < 0.01 indicated statistically significant differences.

**Fig 12:**
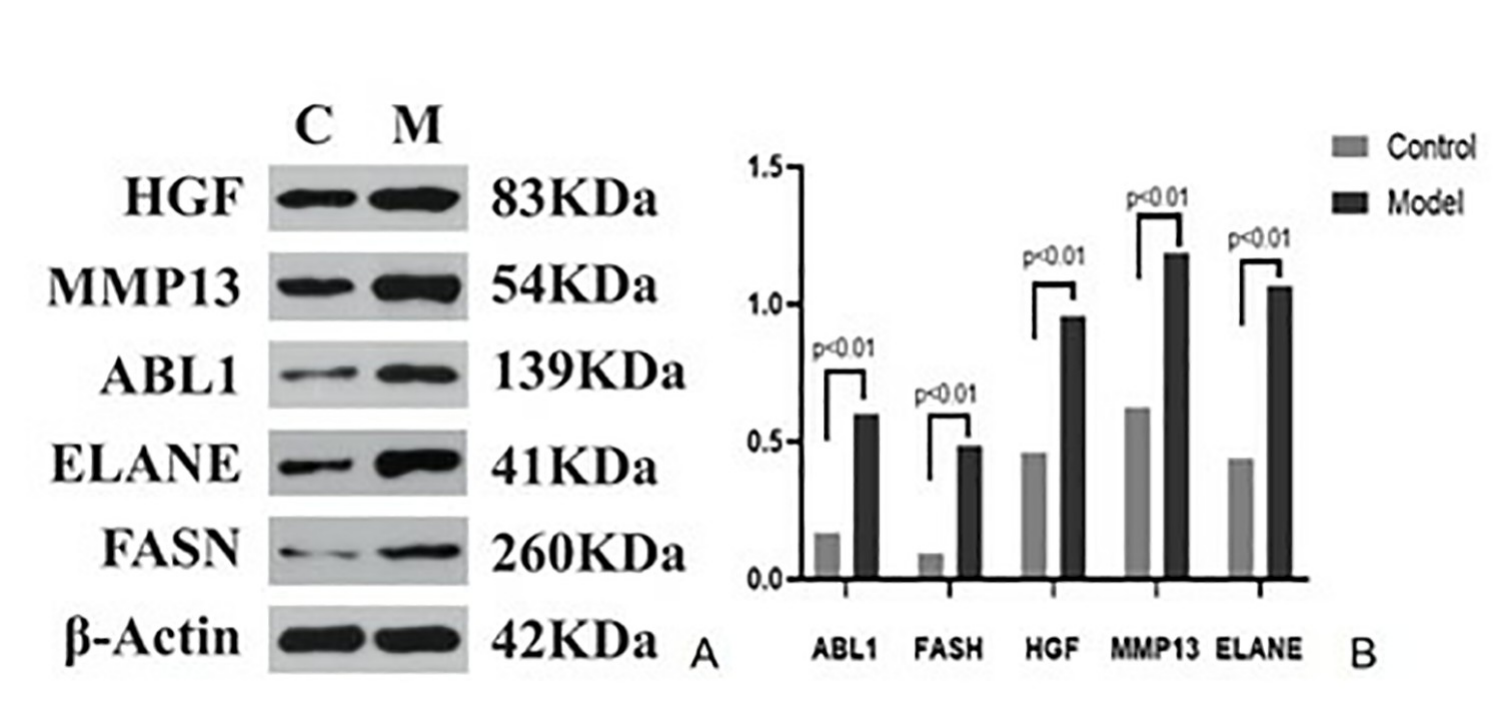
Expression levels of spinal cord proteins in each group of rats. The data were analyzed using independent sample t-tests and expressed as mean P < 0.01 indicated statistically significant differences.

H&E staining revealed that the overall structure of the spinal cord tissue and axons (black arrows) in the blank group was intact, with no obvious demyelination and structurally complete, tightly wrapped membranes of peripheral nerve bundles. In contrast, the model group exhibited severe spinal cord damage, extensive demyelination (red arrowheads), moderate inflammatory cell infiltration (yellow arrowheads), a large number of vacuoles (green arrowheads) in the nerves, and incomplete membranes of peripheral nerve bundles with partial detachment (blue arrowheads). The above results suggest that the modeling was successful (Fig 10). According to the real-time PCR assay, the mRNA expression levels of *HGF*, *MMP13*, *ABL1*, *ELANE*, and *FASN* were significantly elevated in the model group compared with those in the blank group (P < 0.01, Fig 11). The results of the Western blotting assay further verified that the protein expression levels of *HGF*, *MMP13*, *ABL1*, *ELANE*, and *FASN* in the model group were higher in the model group than those in the blank group (P < 0.01, Fig 12).

## Discussion

LDH is the most prevalent cause of NP, manifesting as pain due to spinal cord compression, which leads to neuronal and glial apoptosis [24]. Currently, the regulatory mechanisms of anoikis-related genes in NP are not well understood. Identifying these genes using machine learning algorithms is crucial for elucidating the pathogenesis of NP and identifying potential diagnostic markers and prevention and treatment regimens.

In this study, we analyzed the GSE124272 dataset from the GEO database using bioinformatics methods, identifying 917 anoikis-related genes. Following the optimization of the screening criteria, 86 genes associated with anoikis were selected. GO and KEGG enrichment analyses of the anoikis-related genes using R packages revealed that these DEGs were involved in molecular functions, such as protein serine kinase activity and protein serine/threonine kinase activity, and that they were enriched in signaling pathways, such as the PI3K-Akt pathway, VEGF pathway, and TNF pathway.

Six key anoikis-related genes, namely *HGF*, *MMP13*, *ABL1*, *ELANE*, *FASN*, and *LINC00324*, were identified by examining the overlapping results of LASSO regression and SVM. The diagnostic value of these key genes was subsequently verified by the ROC curve, demonstrating excellent diagnostic performance. Research has linked these genes to apoptosis: *HGF* inhibits anoikis in pancreatic cancer cells *via* the PI3K pathway [25], and *MMP13* reduces anoikis through neural/neuroglial antigen 2 (NG2) shedding [26]. Furthermore, *ABL1* is highly expressed in colorectal cancer cells, and the absence of *ABL1* inhibits cell proliferation and abnormally elevated apoptosis in two cell lines, SW480 and HCT-116, in models of colorectal cancer [27]. It was found that transient and regulated expression of mutant *ELANE* leads to cell death by accelerating apoptosis [28]. *FASN* induces apoptosis by promoting cell proliferation and migration. In osteosarcoma cells, the upregulation of *FASN* enhances cell anoikis resistance, and the knockdown of *FASN* significantly increases the rate of anoikis [29]. In patients with leukemia, the overexpression of *LINC00324* promotes leukemia cell proliferation and inhibits apoptosis [30].

Experimental validation using Western blotting and PCR assays revealed significantly significant differences in the expression levels of *HGF*, *MMP13*, *ABL1*, *ELANE*, and *FASN* between the blank and model groups (P < 0.01).

The sensitization of injury receptors by the immune system involves complement cells, immune cells, glial cells, cytokines, and chemokines, whereas nerve injury is commonly involved in the pathological generation of pain through neuro-immune interactions and neuroinflammation. Immune cells play an important role in promoting the development of neuroinflammation and NP [31]. Therefore, we performed a GSEA and immunoassay for the six key anoikis-related genes. The results showed significant differences in the immune cell infiltration of CD8 + T cells, γδm cells, and neutrophils between the model and blank groups, as well as correlations with 16 immune cell types. Specifically, *MMP13* and Macrophages M0 were significantly negatively correlated with plasma cells and significantly positively correlated with monocytes. These findings suggest that the identified genes play a regulatory role in the immune response during the pathogenesis of NP.

Further analysis on the Enrichr platform identified *ABL1* and *FASN* as the most relevant TFs linked to key anoikis-related genes involved in LDH-associated NP. Furthermore, four potential drugs, valdecoxib, benzocaine, netilmicin, and orphenadrine, were predicted to have anti-inflammatory and analgesic effects in individuals with LDH-associated NP. Valdecoxib is widely applied as a selective COX-2 inhibitor and a nonsteroidal anti-inflammatory drug (NSAID) for pain relief in individuals with osteoarthritis and rheumatoid arthritis [32]. Valdecoxib has been reported to ameliorate lipid-induced insulin resistance in skeletal muscle by inhibiting inflammation and endoplasmic reticulum stress [33]. Parecoxib, a COX 2 inhibitor, and its active metabolite valdecoxib have been shown to reduce NP levels after sciatic nerve ligation in rats [34]. Benzocaine is an anesthetic agent used primarily for disorders associated with oral ulcers, earaches, and dental complications. Studies have shown that benzocaine is significantly better than lidocaine for analgesia in a variety of minor oral surgical procedures in children, effectively relieving surgery-related pain [35]. Netilmicin is a common antibiotic in clinical practice. The combination of moxifloxacin and netilmicin in patients with drug-resistant tuberculosis has a higher effectiveness and safety profile compared with moxifloxacin alone. This approach may lead to fewer inflammatory factors and improved immune function in patients with drug-resistant tuberculosis [36]. In addition, studies have shown that netilmicin has significant advantages in treating gram-negative bacterial meningitis [37]. However, whether netilmicin can improve NP-related neuroinflammation remains to be confirmed. Orphenadrine, a central-acting skeletal muscle relaxant, was an early treatment for Parkinson’s disease. Some researchers have found that its analgesic mechanism may be related to sodium channel blockade [38]. Endogenous circulating analogs of orphenadrine were found to potentially play a role in the prevention of neuronal sensitization in a rat model of CCI by attenuating the hyperexcitability of a harmful stimulus [39].

This study has several limitations. First, the study was conducted based on the GEO database, which included limited samples.This small sample size may reduce the generalizability of the results. Thus, future studies with more patients are needed to validate our findings. Additionally, the use of the GEO database represents a limitation, as it does not account for the clinical features of the patients whose samples were analyzed, such as age, duration of NP, and comorbidities. Finally, although key genes were validated through several tests, further validation of potential therapeutic agents is needed. In the future, we plan to conduct additional *in vitro* and *in vivo* experiments to validate the diagnostic and therapeutic efficacy of the identified key genes and medications for NP.

## Conclusions

This study elucidated the diagnostic significance of anoikis in patients with NP. Through a series of bioinformatics analyses, we ultimately predicted that six key genes, including *HGF*, *MMP13*, *ABL1*, *ELANE*, *FASN*, and *LINC00324*, have certain diagnostic value for NP, which were experimentally validated. These findings provide a reference for further exploring the diagnostic biomarkers and pathogenesis of NP.

## Supporting information

S1 File Figs 1–11. (RAR)

S1 Raw data (PDF)

## References

[1] Colloca L, Ludman T, Bouhassira D, Baron R, Dickenson AH, Yarnitsky D, et al. Neuropathic pain. Nat Rev Dis Primers. 2017;3:17002. doi: 10.1038/nrdp.2017.2 28205574 PMC5371025

[2] Lorio M, Kim C, Araghi A, Inzana J, Yue JJ. International Society for the Advancement of Spine Surgery Policy 2019-Surgical Treatment of Lumbar Disc Herniation with Radiculopathy. Int J Spine Surg. 2020;14(1):1–17. doi: 10.14444/7001 32128297 PMC7043814

[3] Orhurhu MS, Chu R, Claus L, Roberts J, Salisu B, Urits I, et al. Neuropathic Pain and Sickle Cell Disease: a Review of Pharmacologic Management. Curr Pain Headache R. 2020;24(9):52. doi: 10.1007/s11916-020-00885-5 32705357

[4] van Hecke O, Austin SK, Khan RA, Smith BH, Torrance N. Neuropathic pain in the general population: a systematic review of epidemiological studies. Pain. 2014;155(4):654–62. doi: 10.1016/j.pain.2013.11.013 24291734

[5] Taddei ML, Giannoni E, Fiaschi T, Chiarugi P. Anoikis: an emerging hallmark in health and diseases. J Pathol. 2012;226(2):380–93. doi: 10.1002/path.3000 21953325

[6] Bao Y, Wang L, Liu H, Yang J, Yu F, Cui C, et al. A Diagnostic Model for Parkinson’s Disease Based on Anoikis-Related Genes. Mol Neurobiol. 2024;61(6):3641–56. doi: 10.1007/s12035-023-03753-6 38001358

[7] Zhang D, Zhou X, Zhang K, Yu Y, Cui SW, Nie S. Glucomannan from Aloe vera gel maintains intestinal barrier integrity via mitigating anoikis mediated by Nrf2-mitochondria axis. Int J Biol Macromol. 2023;235:123803. doi: 10.1016/j.ijbiomac.2023.123803 36841393

[8] Ikeda T, Nakamura K, Sato T, Kida T, Oku H. Involvement of Anoikis in Dissociated Optic Nerve Fiber Layer Appearance. Int J Mol Sci. 2021;22(4). doi: 10.3390/ijms22041724 33572210 PMC7914697

[9] Bunk EC, Konig HG, Bernas T, Engel T, Henshall DC, Kirby BP, et al. BH3-only proteins BIM and PUMA in the regulation of survival and neuronal differentiation of newly generated cells in the adult mouse hippocampus. Cell Death Dis. 2010;1(1):e15. doi: 10.1038/cddis.2009.13 21364616 PMC3039291

[10] Carreras FJ. Lessons from glaucoma: rethinking the fluid-brain barriers in common neurodegenerative disorders. Neural Regen Res. 2019;14(6):962–6. doi: 10.4103/1673-5374.249215 30762000 PMC6404483

[11] Liao C, Zhou H, Chen H, Cheng G, Li S, Ma F, et al. DUSP8/TAK1 signaling mediates neuropathic pain through regulating neuroinflammation and neuron death in a spinal nerve ligation (SNL) rat model. Int Immunopharmacol. 2022;113(Pt A):109284. doi: 10.1016/j.intimp.2022.109284 36279673

[12] Li L, Li T, Qu X, Sun G, Fu Q, Han G. Stress/cell death pathways, neuroinflammation, and neuropathic pain. Immunol Rev. 2024;321(1):33–51. doi: 10.1111/imr.13275 37688390

[13] Wang Y, Dai G, Jiang L, Liao S, Xia J. Microarray analysis reveals an inflammatory transcriptomic signature in peripheral blood for sciatica. Bmc Neurol. 2021;21(1):50. doi: 10.1186/s12883-021-02078-y 33535986 PMC7856817

[14] Wang Y, Dai G, Li L, Liu L, Jiang L, Li S, et al. Transcriptome signatures reveal candidate key genes in the whole blood of patients with lumbar disc prolapse. Exp Ther Med. 2019;18(6):4591–602. doi: 10.3892/etm.2019.8137 31777557 PMC6862187

[15] Stelzer G, Rosen N, Plaschkes I, Zimmerman S, Twik M, Fishilevich S, et al. The GeneCards Suite: From Gene Data Mining to Disease Genome Sequence Analyses. Curr Protoc Bioinformatics. 2016;54:1–30. doi: 10.1002/cpbi.5 27322403

[16] Chen Y, Huang W, Ouyang J, Wang J, Xie Z. Identification of Anoikis-Related Subgroups and Prognosis Model in Liver Hepatocellular Carcinoma. Int J Mol Sci. 2023;24(3). doi: 10.3390/ijms24032862 36769187 PMC9918018

[17] Zhang H, Meltzer P, Davis S. RCircos: an R package for Circos 2D track plots. Bmc Bioinformatics. 2013;14:244. doi: 10.1186/1471-2105-14-244 23937229 PMC3765848

[18] Ritchie ME, Phipson B, Wu D, Hu Y, Law CW, Shi W, et al. limma powers differential expression analyses for RNA-sequencing and microarray studies. Nucleic Acids Res. 2015;43(7):e47. doi: 10.1093/nar/gkv007 25605792 PMC4402510

[19] Huang S, Cai N, Pacheco PP, Narrandes S, Wang Y, Xu W. Applications of Support Vector Machine (SVM) Learning in Cancer Genomics. Cancer Genom Proteom. 2018;15(1):41–51. doi: 10.21873/cgp.20063 29275361 PMC5822181

[20] Cheng C, Hua ZC. Lasso Peptides: Heterologous Production and Potential Medical Application. Front Bioeng Biotech. 2020;8:571165. doi: 10.3389/fbioe.2020.571165 33117783 PMC7549694

[21] Park SH, Goo JM, Jo CH. Receiver operating characteristic (ROC) curve: practical review for radiologists. Korean J Radiol. 2004;5(1):11–8. doi: 10.3348/kjr.2004.5.1.11 15064554 PMC2698108

[22] Subramanian A, Tamayo P, Mootha VK, Mukherjee S, Ebert BL, Gillette MA, et al. Gene set enrichment analysis: a knowledge-based approach for interpreting genome-wide expression profiles. P Natl Acad Sci Usa. 2005;102(43):15545–50. doi: 10.1073/pnas.0506580102 16199517 PMC1239896

[23] Kuleshov MV, Jones MR, Rouillard AD, Fernandez NF, Duan Q, Wang Z, et al. Enrichr: a comprehensive gene set enrichment analysis web server 2016 update. Nucleic Acids Res. 2016;44(W1):W90–7. doi: 10.1093/nar/gkw377 27141961 PMC4987924

[24] Li ZY, Zhou AF, Li G, Zhou LY, Pu PM, Zhu K, et al. Chronic spinal cord compression associated with intervertebral disc degeneration in SPARC-null mice. Neural Regen Res. 2023;18(3):634–42. doi: 10.4103/1673-5374.350210 36018188 PMC9727435

[25] Watanabe S, Kishimoto T, Yokosuka O. Hepatocyte growth factor inhibits anoikis of pancreatic carcinoma cells through phosphatidylinositol 3-kinase pathway. Pancreas. 2011;40(4):608–14. doi: 10.1097/MPA.0b013e318214fa6c 21499215

[26] Joo NE, Miao D, Bermudez M, Stallcup WB, Kapila YL. Shedding of NG2 by MMP-13 attenuates anoikis. Dna Cell Biol. 2014;33(12):854–62. doi: 10.1089/dna.2014.2399 25166220 PMC4248241

[27] Liu Y, Cao J, Zhu YN, Ma Y, Murtaza G, Li Y, et al. C1222C Deletion in Exon 8 of ABL1 Is Involved in Carcinogenesis and Cell Cycle Control of Colorectal Cancer Through IRS1/PI3K/Akt Pathway. Front Oncol. 2020;10:1385. doi: 10.3389/fonc.2020.01385 32850446 PMC7433659

[28] Makaryan V, Kelley M, Fletcher B, Archibald I, Poulsen T, Dale D. Comparison of Gene Editing versus a Neutrophil Elastase Inhibitor as Potential Therapies for ELANE Neutropenia. J Cell Immunol. 2022;4(1):19–28. doi: 10.33696/immunology.4.129 36052149 PMC9431957

[29] Sun T, Zhong X, Song H, Liu J, Li J, Leung F, et al. Anoikis resistant mediated by FASN promoted growth and metastasis of osteosarcoma. Cell Death Dis. 2019;10(4):298. doi: 10.1038/s41419-019-1532-2 30931932 PMC6443797

[30] Sun GK, Xu ZJ, Nan FY, Tang LJ, Yao DM. Dysregulation of LINC00324 associated with methylation facilitates leukemogenesis in de novo acute myeloid leukemia. Int J Lab Hematol. 2022;44(3):567–75. doi: 10.1111/ijlh.13809 35218157

[31] Fiore NT, Keating BA, Chen Y, Williams SI, Moalem-Taylor G. Differential Effects of Regulatory T Cells in the Meninges and Spinal Cord of Male and Female Mice with Neuropathic Pain. Cells-Basel. 2023;12(18). doi: 10.3390/cells12182317 37759539 PMC10527659

[32] Edwards JE, McQuay HJ, Moore AR. Efficacy and safety of valdecoxib for treatment of osteoarthritis and rheumatoid arthritis: systematic review of randomised controlled trials. Pain. 2004;111(3):286–96. doi: 10.1016/j.pain.2004.07.004 15363872

[33] lipid-induced skeletal muscle insulin resistance via simultaneous suppression of inflammation and endoplasmic reticulum stress. Biochem Pharmacol. 2021;188:114557. 10.1016/j.bcp.2021.114557. 33844985

[34] Schroder H, Hollt V, Becker A. Parecoxib and its metabolite valdecoxib directly interact with cannabinoid binding sites in CB1-expressing HEK 293 cells and rat brain tissue. Neurochem Int. 2011;58(1):9–13. doi: 10.1016/j.neuint.2010.10.018 21073910

[35] John NE, Erakkodan R, Sreelakshmi N. Efficacy of benzocaine and lidocaine bioadhesive patches in various minor oral surgical procedures in children—An evaluative study. Indian J Dent Res. 2023;34(2):145–9. doi: 10.4103/ijdr.ijdr_192_21 37787201

[36] Wang L, Zhang Y, Chen L, Zhang G, Zhu H. Effectiveness and safety of co-administration of moxifloxacin with netilmicin in drug-resistant tuberculosis patients, and its impact on inflammatory factors and immune function. Trop J Pharm Res. 2021;20(5):1085–90. 10.4314/tjpr.v20i5.29.

[37] Scheld WM, Brown RJ, Sande MA. Comparison of netilmicin with gentamicin in the therapy of experimental Escherichia coli meningitis. Antimicrob Agents Ch. 1978;13(6):899–904. doi: 10.1128/AAC.13.6.899 354518 PMC352359

[38] Desaphy JF, Dipalma A, De Bellis M, Costanza T, Gaudioso C, Delmas P, et al. Involvement of voltage-gated sodium channels blockade in the analgesic effects of orphenadrine. Pain. 2009;142(3):225–35. doi: 10.1016/j.pain.2009.01.010 19217209

[39] Biella GE, Groppetti A, Novelli A, Fernandez-Sanchez MT, Manfredi B, Sotgiu ML. Neuronal sensitization and its behavioral correlates in a rat model of neuropathy are prevented by a cyclic analog of orphenadrine. J Neurotraum. 2003;20(6):593–601. 10.1089/089771503767168519. 12906743

