# Supplementary figures and images for: Prediction and validation of anoikis-related genes in neuropathic pain using machine learning
Source: PLoS One. 2025 Feb 27;20(2):e0314773. doi: 10.1371/journal.pone.0314773 (PMC11867322; doi:10.1371/journal.pone.0314773)

# First experiment

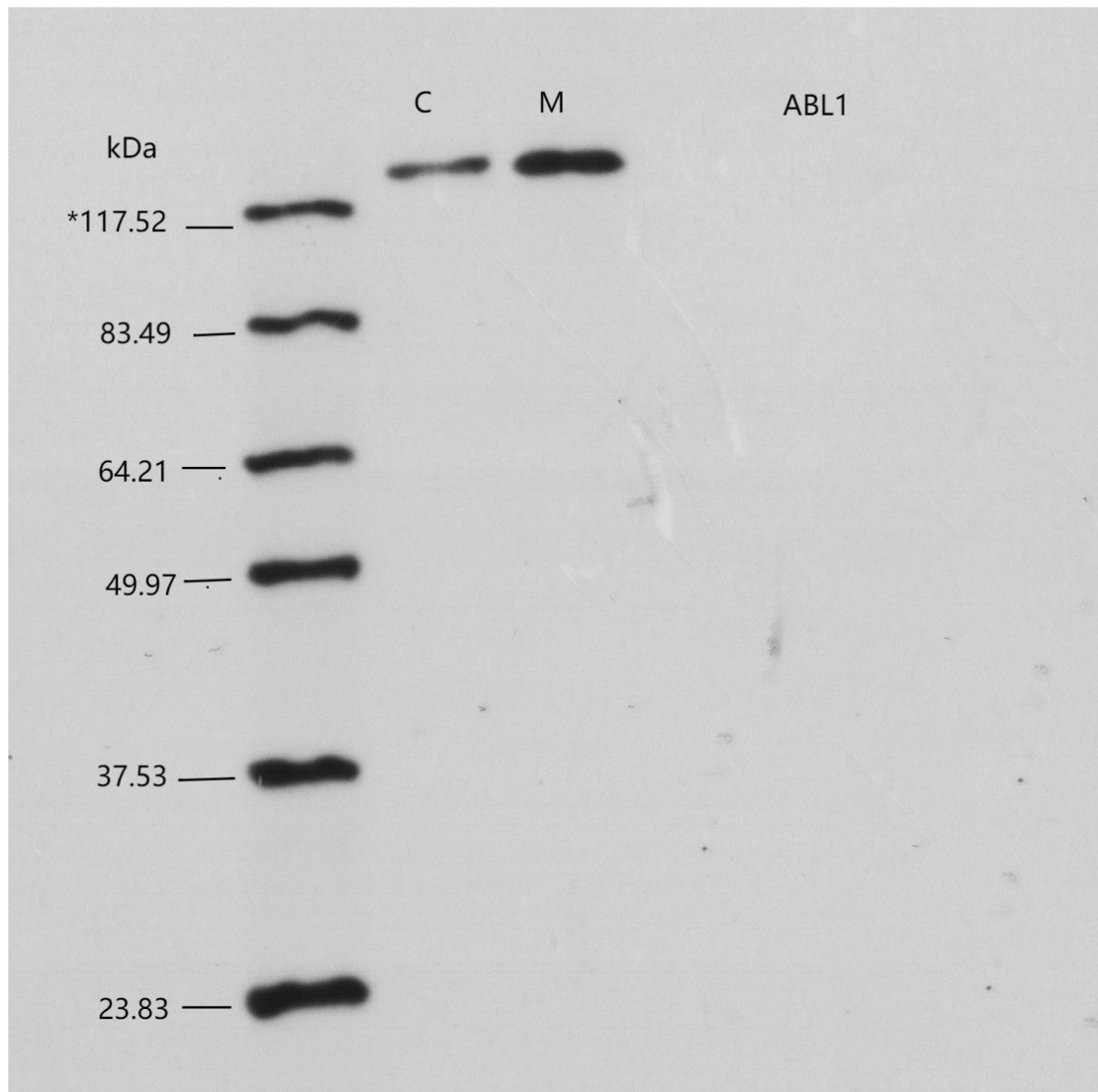

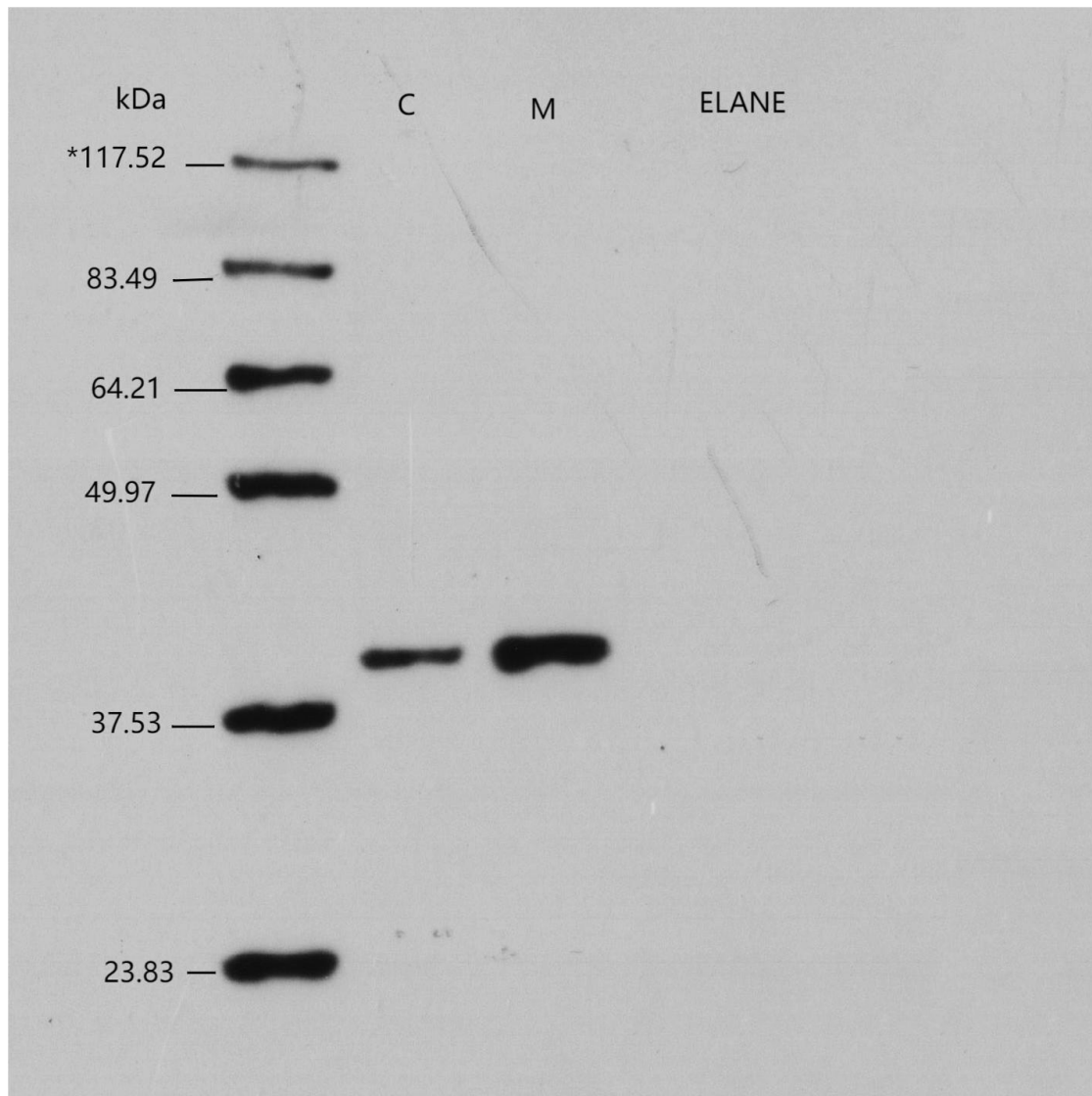

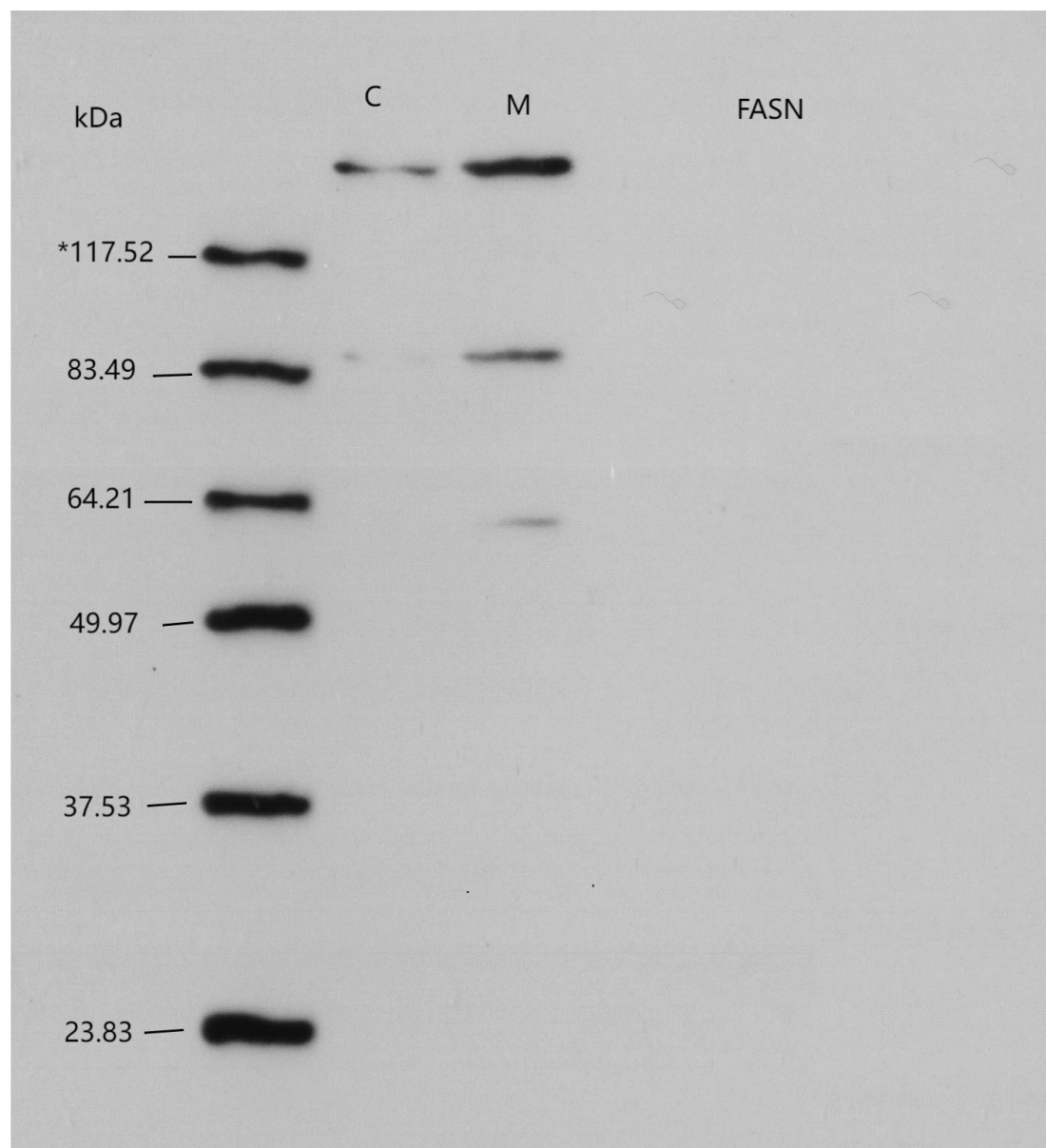

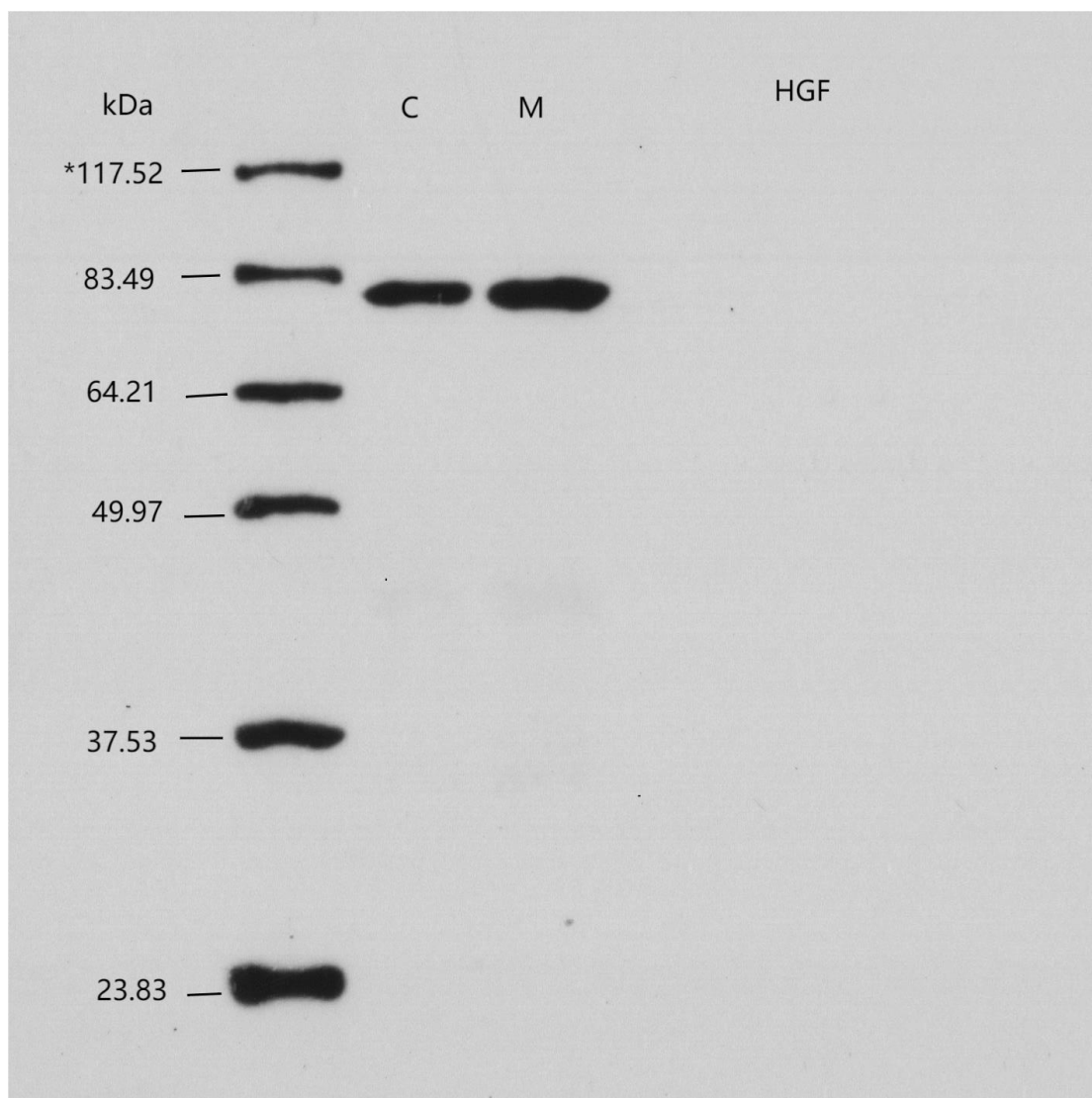

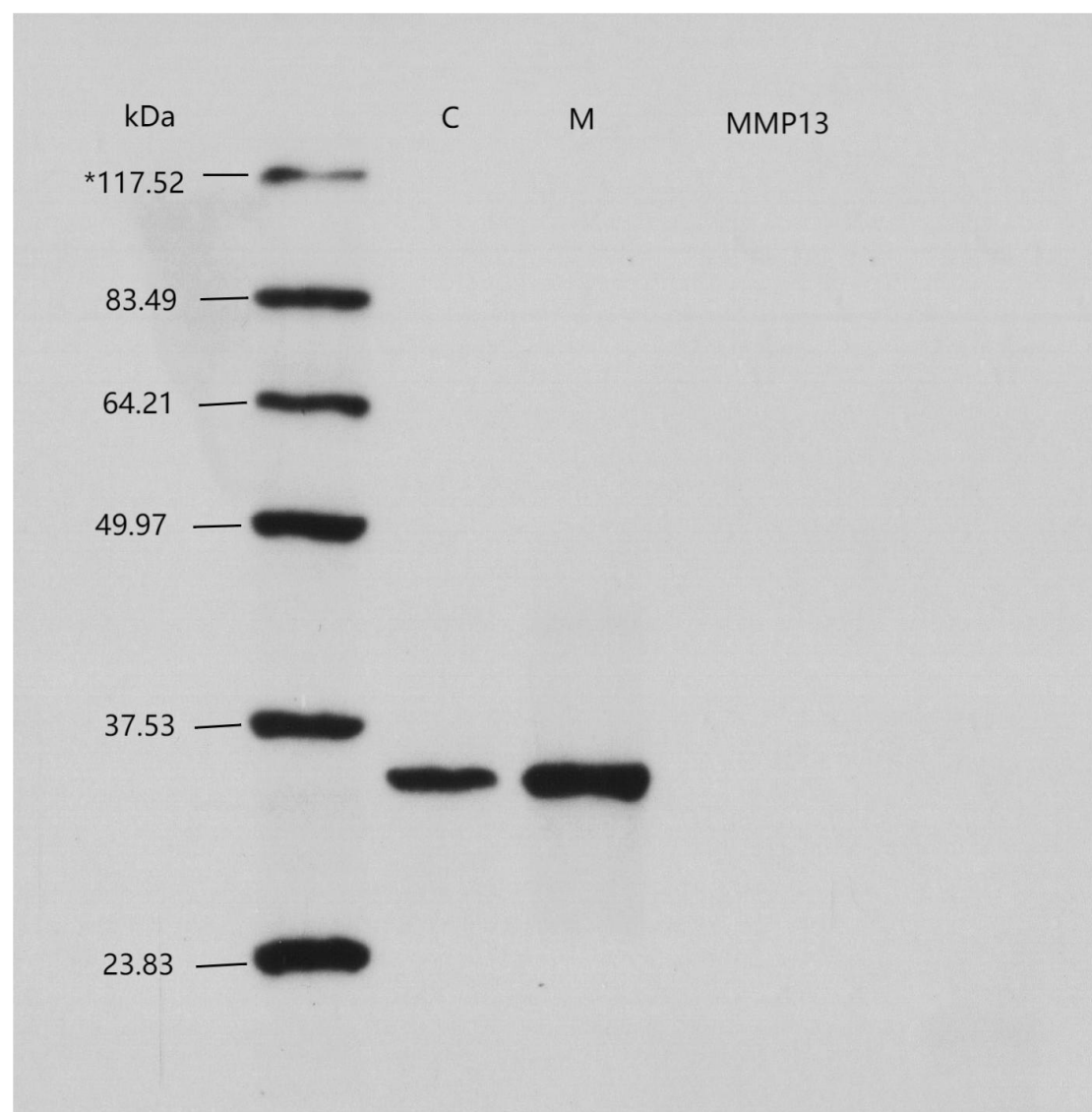

Repeated experimental results twice

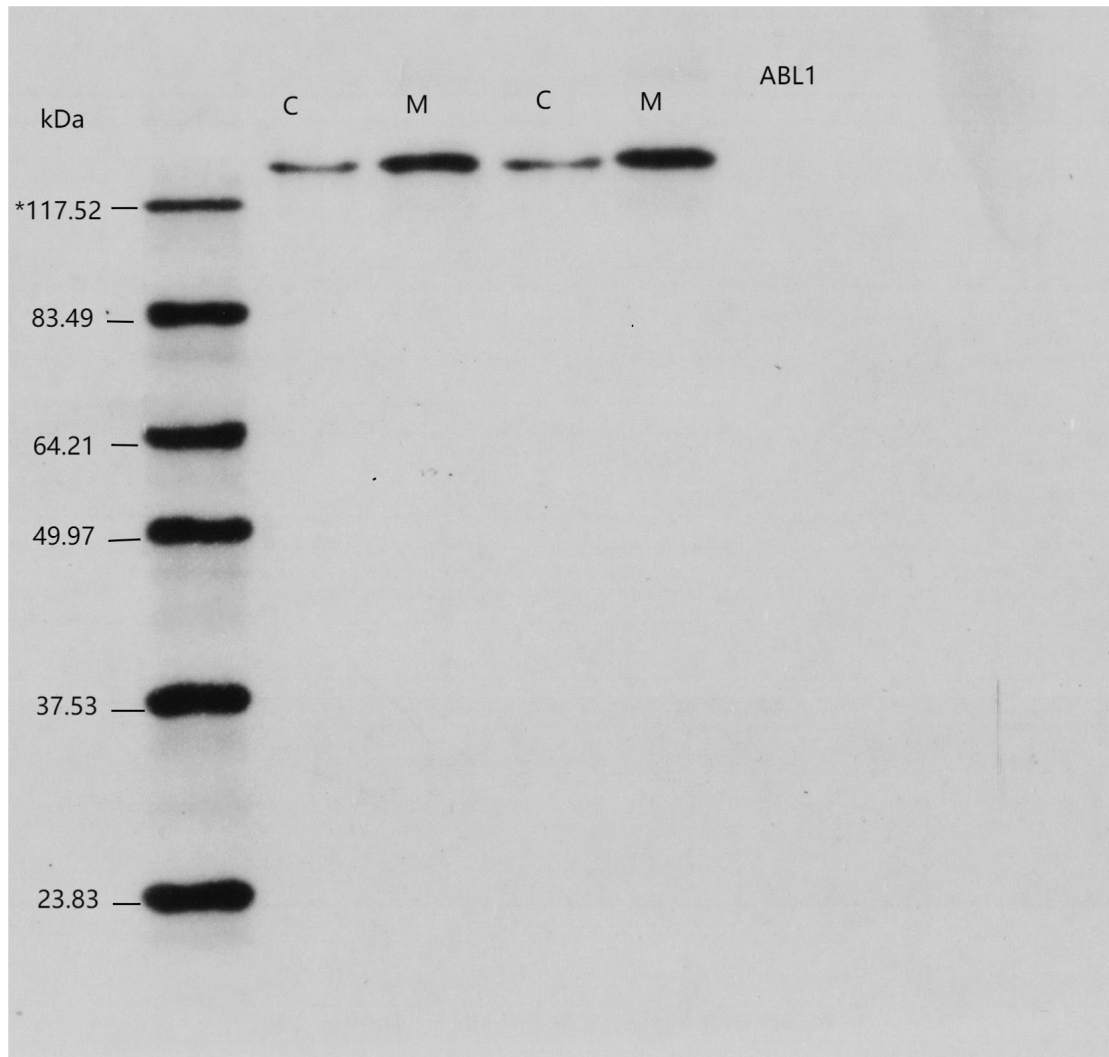

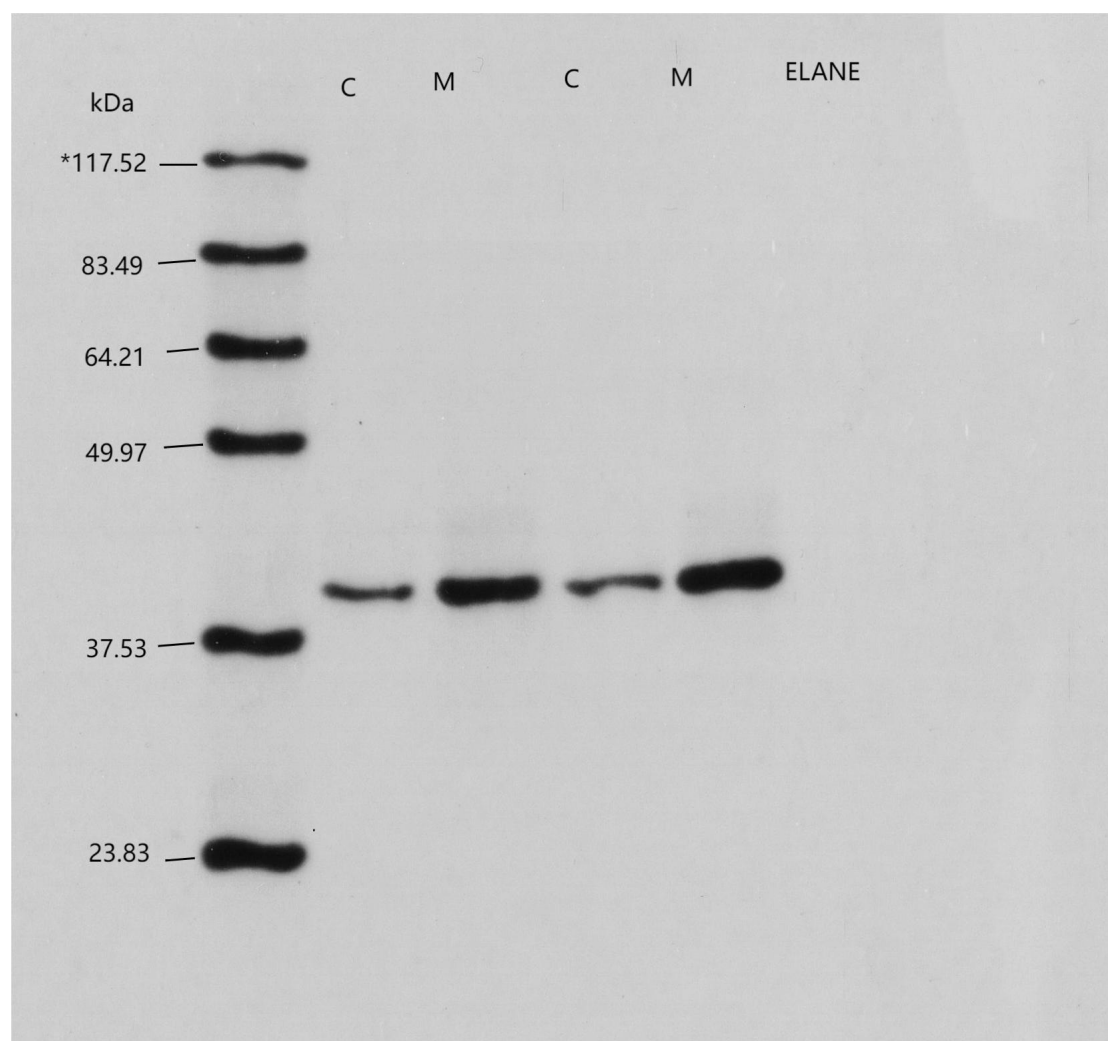

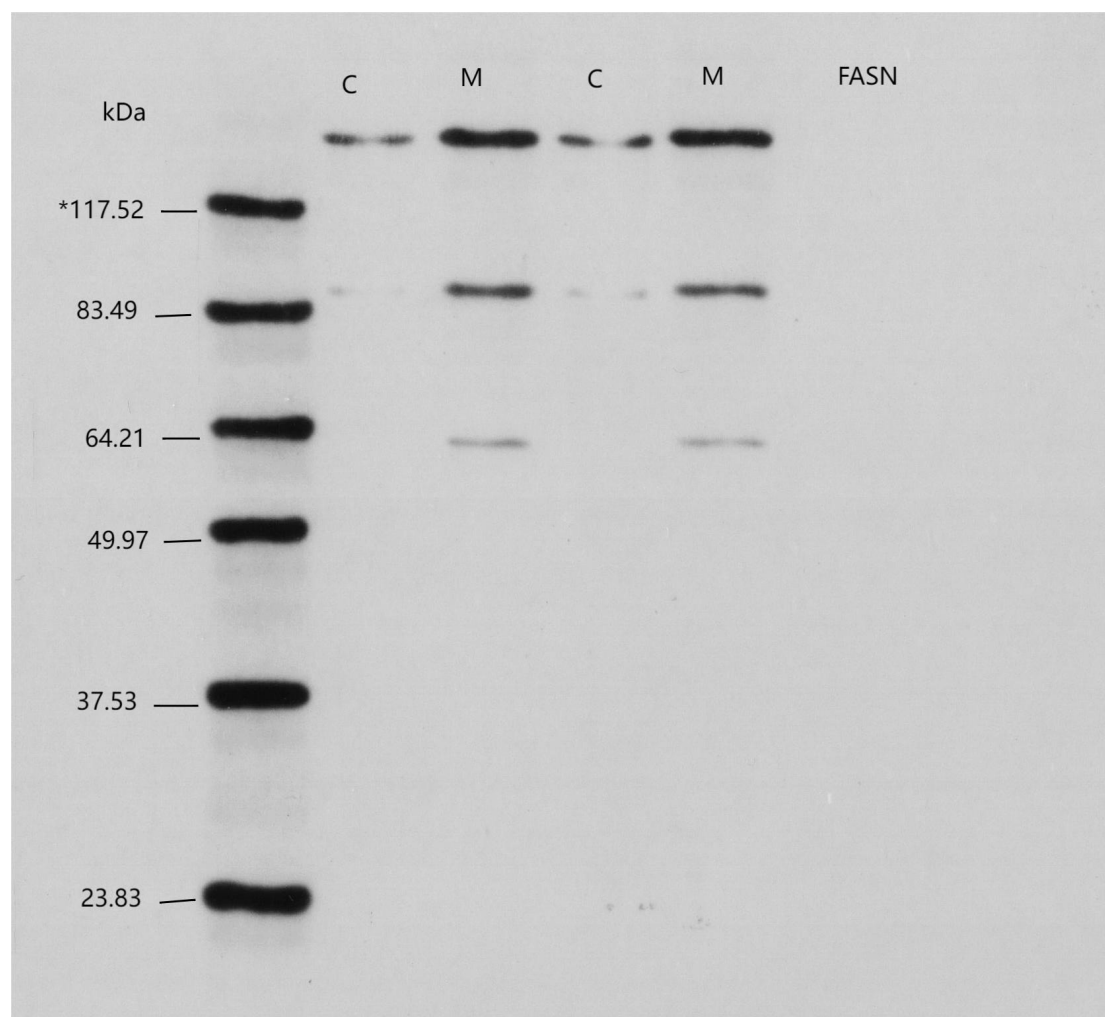

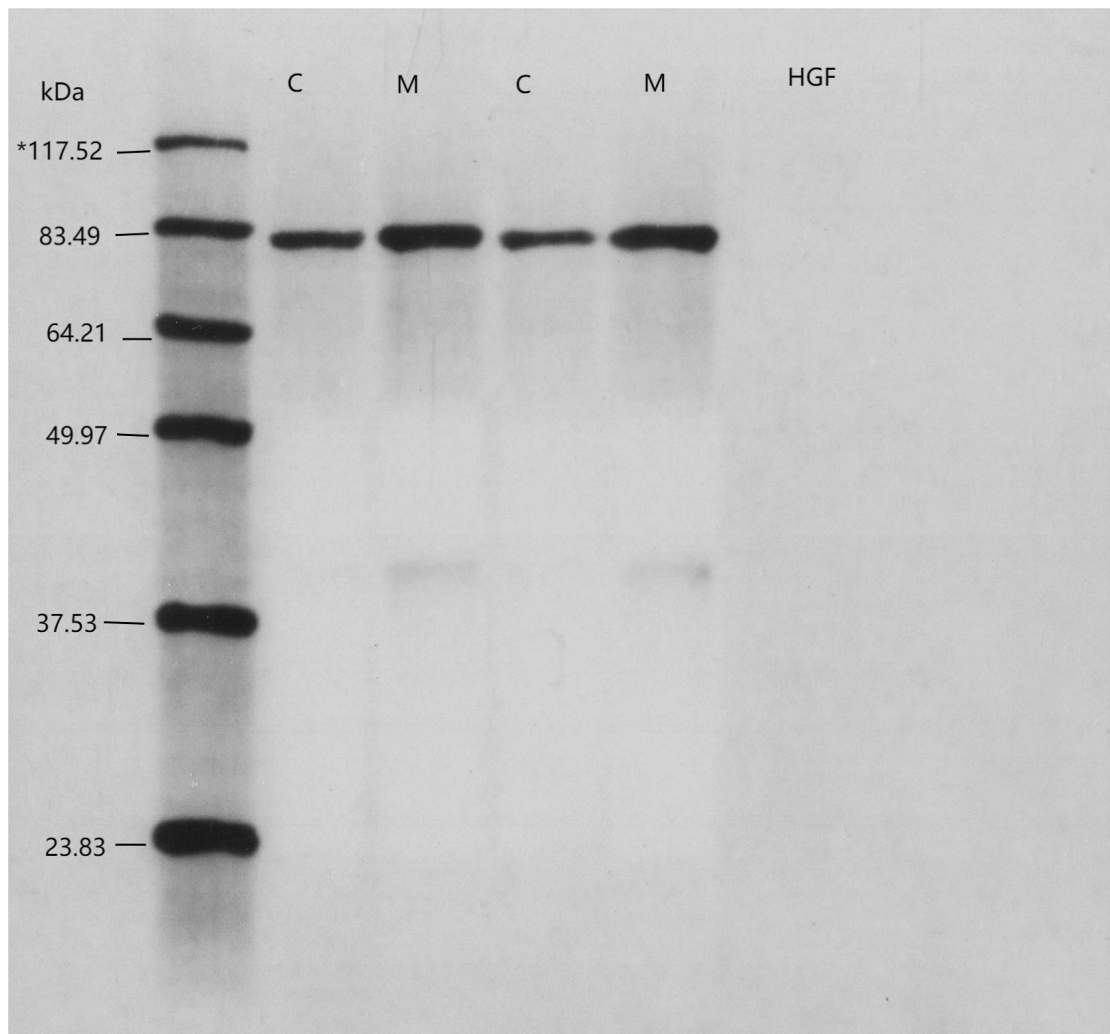

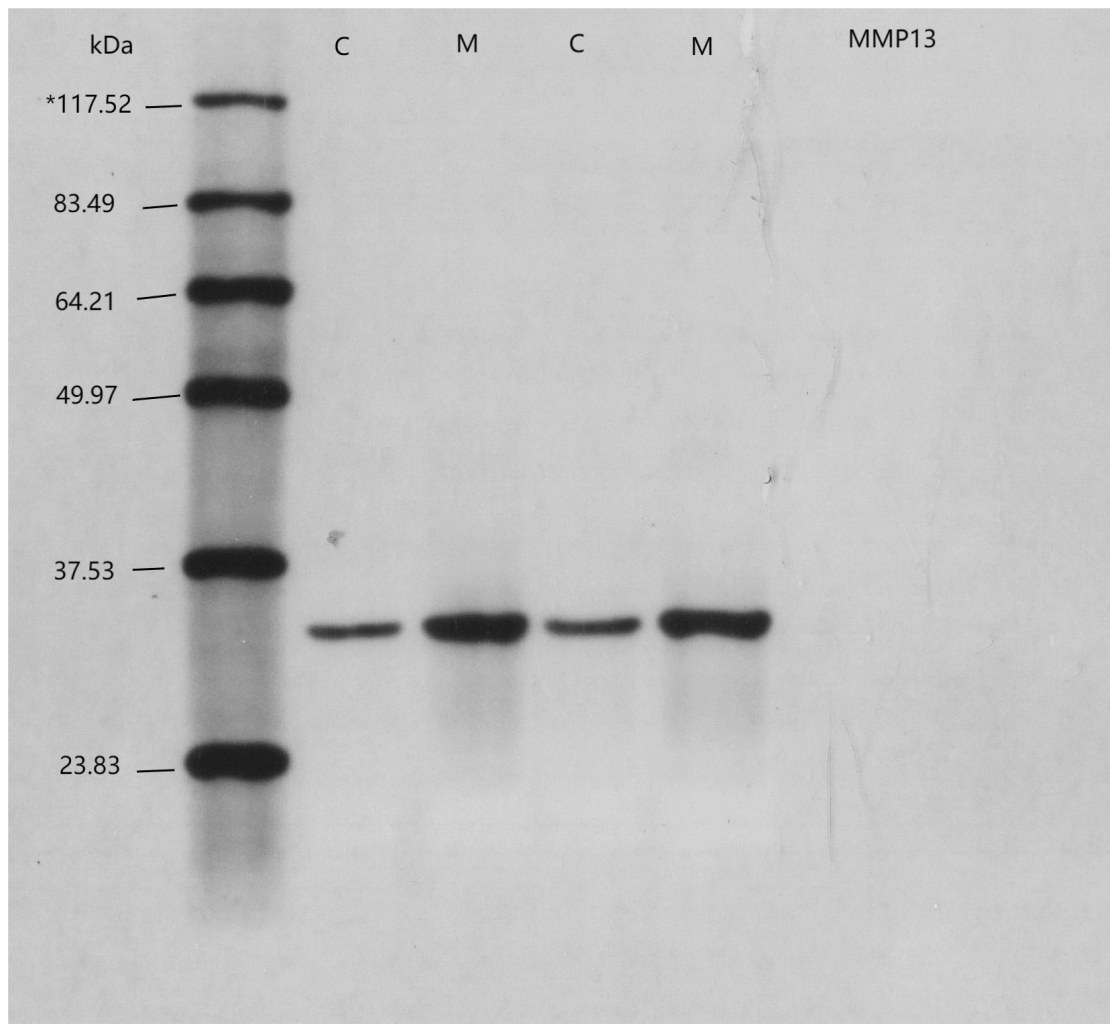

Supplement: S1 Raw data — (PDF) [file pone.0314773.s002.pdf]
